# Implementation of a patient blood management in an Italian City Hospital: is it effective in reducing the use of red blood cells?

**DOI:** 10.1007/s13304-022-01409-z

**Published:** 2022-10-30

**Authors:** Giorgio Ercolani, Leonardo Solaini, Fabrizio D’Acapito, Claudio Isopi, Carlo Alberto Pacilio, Cinzia Moretti, Vanessa Agostini, Alessandro Cucchetti

**Affiliations:** 1 Department of Medical and Surgical Sciences (DIMEC), University of Bologna, Morgagni-Pierantoni Hospital, Via C. Forlanini 34, Forlì, Italy; 2 General and Oncologic Surgery, Morgagni Pierantoni Hospital, Ausl Romagna, Forlì, Italy; 3 Immunohematology and Transfusion Medicine, Morgagni Pierantoni Hospital, Ausl Romagna, Forlì, Italy; 4 Transfusion Medicine Department, IRCCS-Ospedale Policlinico San Martino, Genoa, Italy

**Keywords:** Transfusion, Major abdominal surgery, Blood management, Anemia, Minimally invasive surgery

## Abstract

To evaluate the effect of patient blood management (PBM) since its introduction, we analyzed the need for transfusion and the outcomes in patients undergoing abdominal surgery for different types of tumor pre- and post-PBM. Patients undergoing elective gastric, liver, pancreatic, and colorectal surgery between 2017 and 2020 were included. The implementation of the PBM program was completed on May 1, 2018. The patients were grouped as follows: those who underwent surgery before the implementation of the program (pre-PBM) versus after the implementation (post-PBM). A total of 1302 patients were included in the analysis (445 pre-PBM vs. 857 post-PBM). The number of transfused patients per year decreased significantly after the introduction of PBM. A strong tendency for a decreased incidence of transfusion was evident in gastric and pancreatic surgery and a similar decrease was statistically significant in liver surgery. With regard to gastric surgery, a single-unit transfusion scheme was used more frequently in the post-PBM group (7.7% vs. 55% after PBM; *p* = 0.049); this was similar in liver surgery (17.6% vs. 58.3% after PBM; *p* = 0.04). Within the subgroup of patients undergoing liver surgery, a significant reduction in the use of blood transfusion (20.5% vs. 6.7%; *p* = 0.002) and a decrease in the Hb trigger for transfusion (8.5, 8.2–9.5 vs. 8.2, 7.7–8.4 g/dl; *p* = 0.039) was reported after the PBM introduction. After the implementation of a PBM protocol, a significant reduction in the number of patients receiving blood transfusion was demonstrated, with a strong tendency to minimize the use of blood products for most types of oncologic surgery.

## Introduction

Blood transfusions are commonly used in general, cardiac, and orthopedic surgery [1–3].

On a large scale, approximately 30% of patients undergoing major abdominal surgery require blood transfusion, with those undergoing hepatic and pancreatic resections at higher risk of receiving blood products during hospitalization [2].

Transfusion may prevent severe complications during uncontrolled bleeding and is considered a life-saving treatment; however, it has negative impacts on the post-operative morbidity and long-term outcomes in oncologic surgical patients [4–7].

By analyzing more than 1200 patients who underwent resection for hepatocellular carcinoma (HCC) or colorectal metastases, we demonstrated that blood transfusion had a negative impact on mortality and that the number of units of packed red cells administered is strongly related to early outcomes; patients receiving two or more units of packed red cells demonstrated a significantly increased risk of post-operative morbidity and mortality [8]. Similarly, Gruttadauria et al. [9], analyzing a cohort of liver resections for colorectal metastases, found that patients who received intraoperative blood transfusions were at higher risk to experience major postoperative complications and, consequently, a longer length of hospital stay.

Patient blood management (PBM) has been introduced in the last decade to reduce the use of blood products and improve tolerance to anemia [10–14]. Most data regarding the relationship between the implementation of PBM and the reduction in the consumption of blood products come from North America [14–17].

To evaluate the effect of PBM since its introduction in our Center in 2018, we analyzed the need for transfusion and the post-operative outcomes in the last 4 years in patients undergoing abdominal surgery for different types of tumor pre- and post-PBM.

## Patients and methods

Patients undergoing elective gastric, liver, pancreatic, and colorectal surgery at Morgagni-Pierantoni Hospital of Forlì from 2017 to 2020 were included in the analysis.

The implementation of the PBM program was completed on May 1, 2018. The patients were grouped as follows: those who underwent surgery before the implementation of the program (from January 1, 2017 to April 30, 2018; pre-PBM) versus after the implementation (from May 1, 2018 to December 31, 2020; post-PBM).

For each patient record, sociodemographic characteristics (age and sex) and illness-related variables (American Society of Anesthesiologists (ASA) score, diagnosis of malignancy) were recorded.

Preoperative Hemoglobin (Hb), platelets count, and INR was also recorded. The type of surgery, the approach (open versus minimally invasive) and postoperative complications were also collected.

Length of hospital stay was defined as the interval between the day of surgery and the day of discharge.

The hemoglobin trigger was defined as the value of hemoglobin below which a transfusion was performed.

### Blood management protocol

In 2017, a multidisciplinary working group was established to promote the most appropriate transfusion practices in accordance with the PBM approaches. Surgeons, anesthesiologists, and specialists in transfusion medicine were involved in this project. A team coordinator was also identified and formally commissioned by hospital management.

The constructed protocol was based on the three pillars of PBM [10, 18] and the blood component transfusion policy was defined in compliance with the current transfusion guidelines for red blood cells, platelets, and plasma.

As shown in Table 1, the protocol included pre-operative, intra-operative, and post-operative precautions to prepare the patient to the procedure and to prevent and manage potential blood losses.

**Table 1:** Summary of the main indications included in the protocol

Pre-operative	Intra-operative	Post-operative
Identify patients with anemia (Hb < 12 g/dl in male and < 13 g/dl in female) at least 30 days before surgery and refer them to Transfusion Medicine	Careful hemostasis and meticulous surgery	Stimulate erythropoiesis and avoid anemia caused by deficiencies
Correct iron, folic acid vitamin B12 deficiencies	Assess the most appropriate approach, using minimally invasive surgery whenever possible	Monitor for postoperative bleeding
Assess the indication for erythropoiesis stimulating agents	Anesthetic blood-sparing strategies (neuraxial anesthesia, permissive hypotension, normothermia…)	Maintain normothermia
Review and management of medications affecting the coagulation	Use hemostatic agents	Avoid/treat infections
Formulate patient-specific plans to minimize blood loss including the use of appropriate blood conservation modalities	Optimize oxygenation, ventilation and cardiac output	

**Fig. 1:**
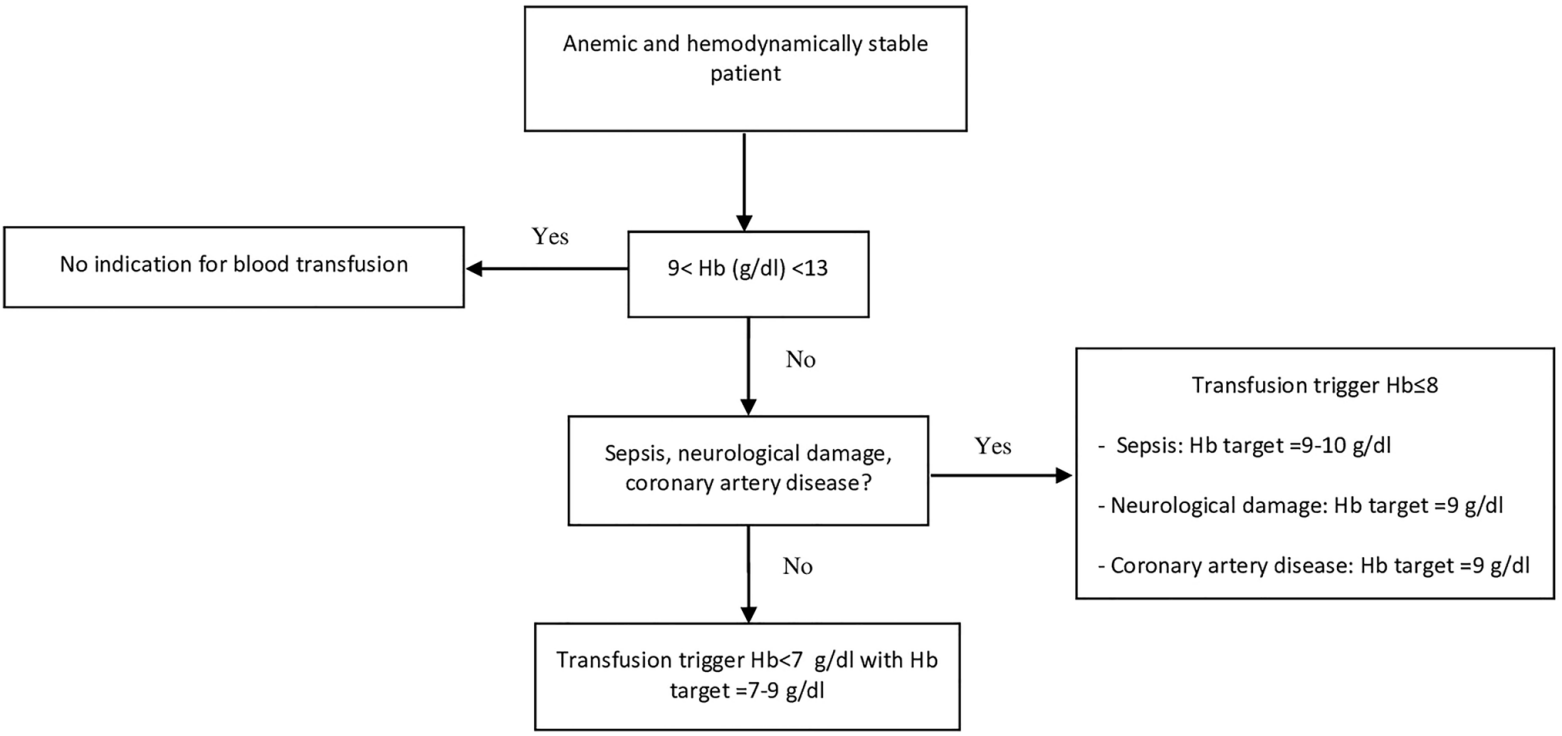
Blood transfusion management algorithm

The restrictive trigger and single-unit blood transfusion practice were part of the protocol to correct peri-operative anemia. Different triggers were modulated and used according to patients’ conditions and comorbidities (Fig. 1). All patients were reassessed before a second unit was requested.

Educational programs and group discussions with surgeons and anesthesiologists were conducted, as were periodic audits of transfusion practices.

### Statistical analysis

Continuous data were shown as median and interquartile range (IQR) and comparisons were performed using the Mann–Whitney *U* test. Fisher exact test was used to compare categorical variables which were presented as frequencies and percentages. Analyses were performed with statistical software for biomedical research (MedCalc® for Windows®, version 10.2.0.0; MedCalc Software, Ostend, Belgium).

## Results

A total of 1302 patients were included in the analysis. In particular, 445 patients were in the pre-PBM group while 857 were in the post-PBM group.

As shown in Table 2, all baseline patients’ characteristics were similar between the groups with the exception of the rate of minimally invasive approaches which was higher in the post-PBM group (140, 31.5% before PBM versus 326, 38% after PBM introduction; *p* = 0.025).

**Table 2:** Patients’ characteristics

	Total (*n* = 1302)	Pre-PBM (*n* = 445)	Post-PBM (*n* = 857)	*p*
Age, median (IQR)	68.8 (58.6–77.1)	67.8 (58.4–76.7)	69.3 (58.7–77.7)	0.267
Sex, *n* (%)				0.906
M	695 (53.4)	239 (53.7)	457 (53.3)	
F	607 (56.6)	206 (46.3)	400 (46.7)	
ASA score, *n* (%)				0.248
> 2	833 (64.0)	275 (61.8)	558 (65.1)	
< 2	469 (36.0)	170 (38.2)	299 (34.9)	
Malignancy, *n* (%)				0.517
Yes	1102 (84.7)	381 (85.6)	721 (84.1)	
No	200 (15.3)	64 (14.4)	136 (15.9)	
Minimally invasive surgery, *n* (%)				0.025
Yes	466 (35.8)	140 (31.5)	326 (38.0)	
No	836 (64.2)	305 (78.5)	531 (72.0)	
Types of procedure, *n* (%)				
Gastric surgery	204 (15.6)	56 (12.6)	148 (16.7)	0.616
Wedge resection	23 (11.4)	7 (12.5)	16 (10.8)	
Subtotal gastrectomy	131 (64.2)	34 (60.7)	97 (65.5)	
Total gastrectomy	50 (24.5)	15 (26.8)	35 (23.6)	
Colorectal surgery	682 (52.4)	255 (57.3)	427 (49.8)	0.667
Right colectomy/ileocecal resection	285 (41.8)	106 (41.6)	179 (41.9)	
Left colectomy/sigmoidectomy	222 (32.5)	89 (34.9)	133 (31.1)	
Total colectomy	4 (0.6)	1 (0.4)	3 (0.7)	
Anterior rectal resection	171	59 (23.1)	112 (26.2)	
Pancreas surgery	153 (11.8)	46 (10.3)	108 (12.6)	0.803
Distal pancreatectomy	43 (28.1)	14 (30.4)	29 (26.8)	
Pancreaticoduodenectomy	53 (34.6)	14 (30.4)	39 (36.1)	
Total pancreatectomy	57 (37.2)	17 (36.9)	40 (37.0)	
Liver surgery	263 (20.2)	83 (18.6)	180 (21.0)	0.343
Lobectomy	22 (8.3)	9 (10.8)	13 (7.2)	
Partial hepatectomy	241 (91.6)	74 (89.2)	167 (92.8)	
Preoperative hemoglobin (g/dl), median (IQR)	13.4 (12.1–14.4)	13.0 (11.6–14.0)	12.9 (11.5–14.1)	0.881
Preoperative platelets count, median (IQR)	231 (188–278)	221 (182–281)	229 (187–280)	0.301
Preoperative INR, median (IQR)	1.0 (0.0–1.1)	1.0 (0.9–1.1)	1.0 (0.0–1.1)	0.872

**Fig. 2:**
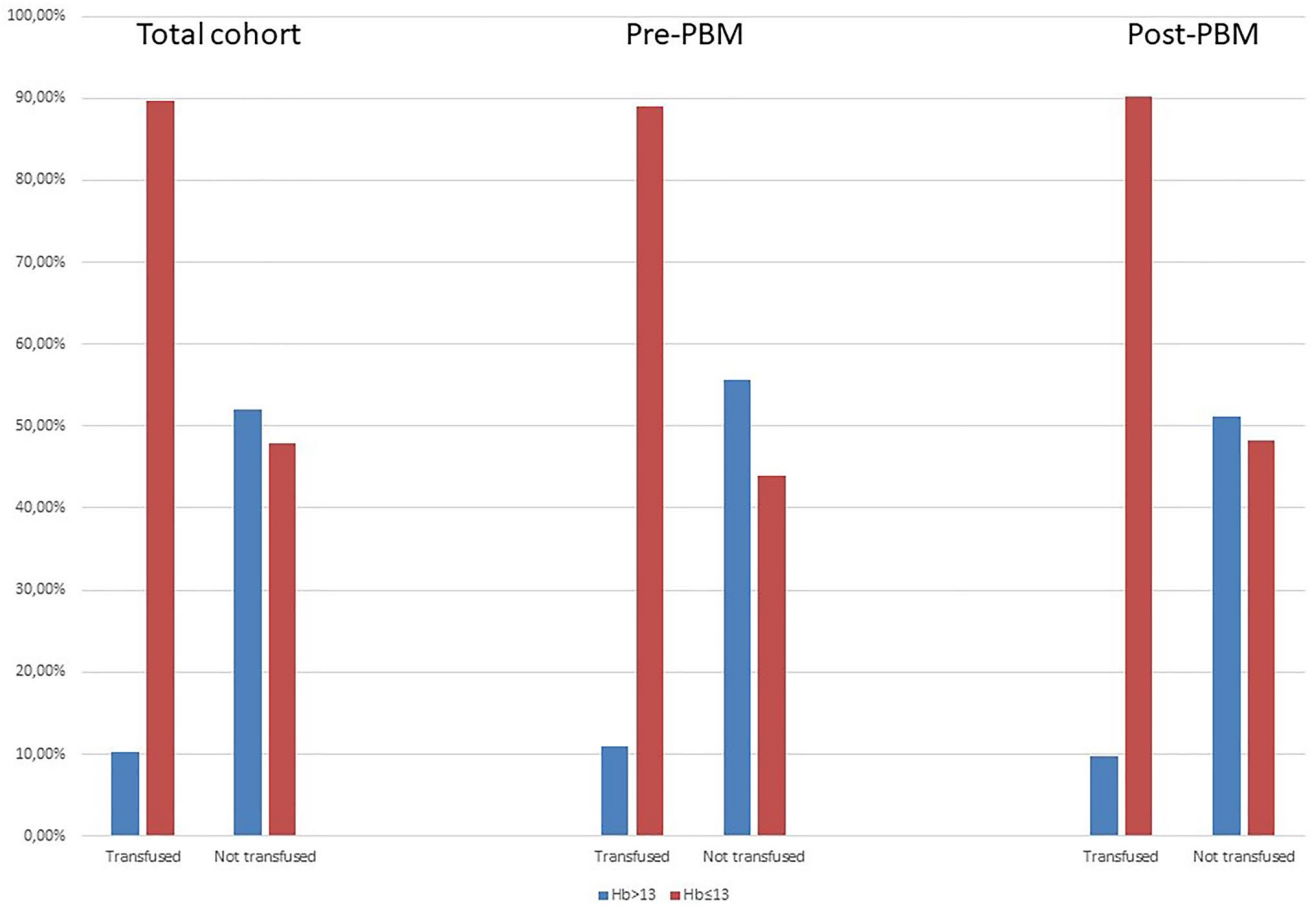
Rate of transfused patients according to preoperative Hb value (less vs. more than 13 gr/dl)

The median preoperative Hb was higher in the patients who did not have postoperative transfusions (13.2, 11.9–14.3 versus 10.4, 9.4–11.7; *p* < 0.0001) and this was seen also within the pre-PBM era (13.2, 12–14.2 versus 10.3, 9.4–11.9; *p* < 0.0001) and in the post-PBM groups (13.1, 11.9–14.3 versus 10.4, 9.4–11.6; *p* < 0.001). The rate of transfused patients according to preoperative Hb value (less or more than 13 gr/dl) is reported in Fig. 2

**Fig. 3:**
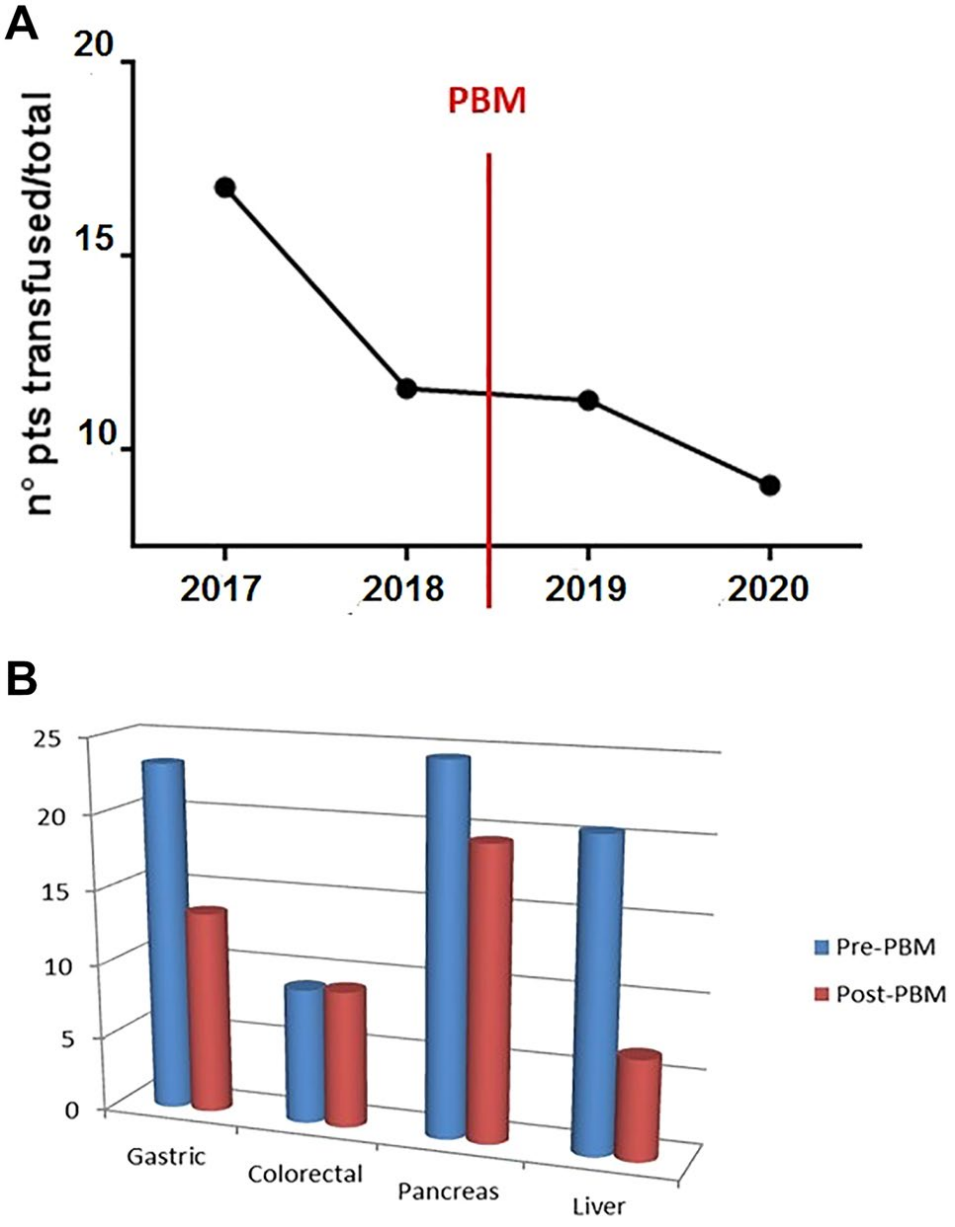
**A** Number of transfused patients per year. **B** Rates of transfusion according to the type of surgery

The number of transfused patients per year decreased significantly after the introduction of PBM, as shown in Fig. 3a. The rate of transfusion according to the type of surgery in each group is shown in Fig. 3b. As shown in Fig. 3b and Table 3, a strong tendency for a decreased incidence of transfusion was evident in gastric and pancreatic surgery and a similar decrease was statistically significant in liver surgery; there was no difference between the two groups of patients requiring colorectal surgery.

**Table 3:** Outcomes

	Total	Pre-PBM	Post-PBM	*p*
**Gastric Surgery**	204	56	148	
Patients transfused	33 (16.2)	13 (23.2)	20 (13.5)	0.138
Hb trigger	8.2 (7.6–8.8)	8.2 (7.6–8.6)	8.3 (7.7–9.1)	0.507
Postoperative complications	54 (26.4)	16 (28.6)	38 (25.7)	0.859
Single unit transfusion scheme				0.049
Yes	9 (4.4)	1 (7.7)	9 (45)	
No	24 (11.8)	12 (92.3)	11 (55)	
Length of hospital stay	10 (8–13)	10 (8–13)	9 (7–14)	0.934
**Colorectal Surgery**	682	255	427	
Patients transfused	62 (9.1)	23 (9.0)	39 (9.1)	1.000
Hb trigger	8.1 (7.6–9.6)	8.3 (7.6–8.6)	8.2 (7.6–8.7)	0.713
Postoperative complications	61 (8.9)	28 (11.0)	33 (7.2)	0.166
Single unit transfusion scheme				1.000
Yes	10 (1.5)	4 (17.4)	6 (15.4)	
No	52 (7.6)	19 (82.6)	33 (84.6)	
Length of hospital stay	7 (6–8)	7 (5–8)	6 (5–8)	0.058
**Pancreas Surgery**	153	45	108	
Patients transfused	32 (20.9)	11 (24.4)	21 (19.4)	0.517
Hb trigger	8.5 (7.8–9.1)	8.6 (7.8–9.1)	8.5 (8.1–8.5)	0.952
Postoperative complications	50 (32.7)	12 (26.7)	38 (35.2)	0.348
Single unit transfusion scheme				0.681
Yes	8 (5.2)	2 (18.2)	6 (28.6)	
No	24 (15.7)	9 (81.8)	15 (71.4)	
Length of hospital stay	11 (9–13)	10 (8–13)	13 (10–14)	< 0.001
**Liver Surgery**	263	83	180	
Patients transfused	29 (11.0)	17 (20.5)	12 (6.7)	0.002
Hb trigger	8.3 (8.1–8.9)	8.5 (8.2–9.5)	8.1 (7.7–8.4)	0.039
Postoperative complications	32 (12.2)	13 (15.7)	19 (10.5)	0.219
Single unit transfusion scheme				0.046
Yes	12 (4.6)	3 (17.6)	7 (58.3)	
No	17 (6.4)	14 (82.3)	5 (41.7)	
Length of hospital stay	8 (6–12)	8 (7–12)	7 (6–11)	0.023

The post-operative outcomes are shown in Table 3. The post-operative complications according to surgery type were similar between the groups.

**Fig. 4:**
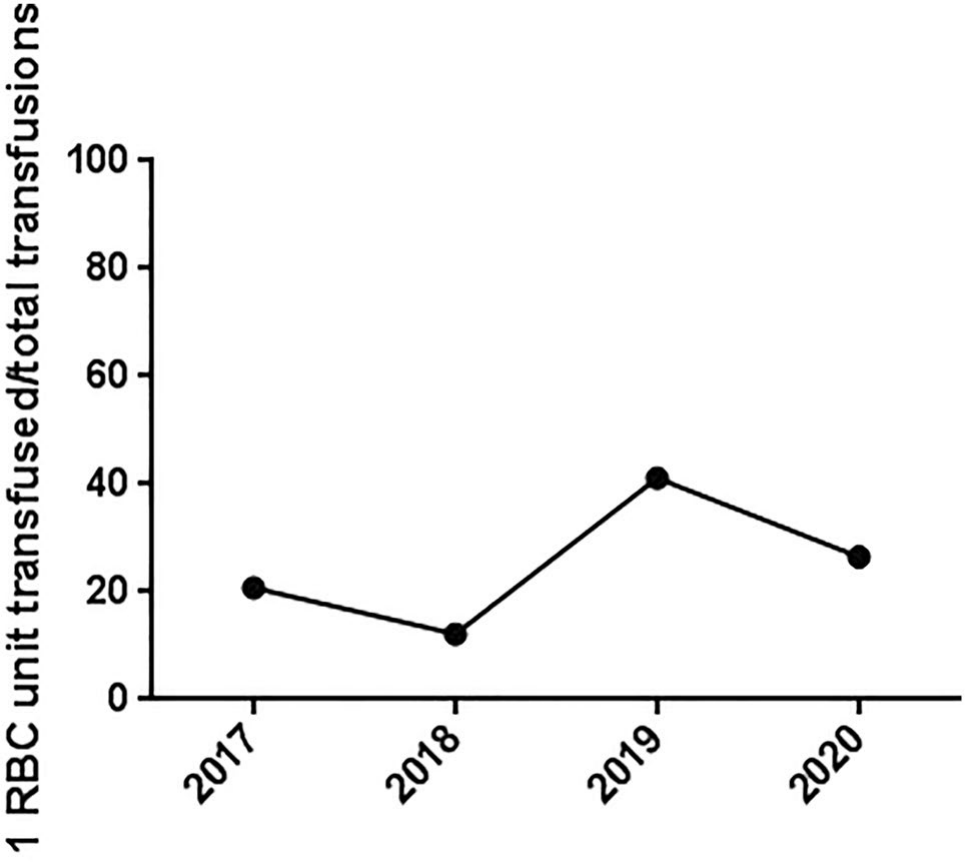
Trend in the rate of adoption of the single-transfusion scheme

With regard to gastric surgery, a single-unit transfusion scheme was used more frequently in the post-PBM group (1, 7.7% vs. 9, 55% after PBM; *p* = 0.049); this was similar in liver surgery (3, 17.6% vs. 7, 58.3% after PBM; *p* = 0.04). A tendency was observed even for pancreatic surgery, although it did not reach statistical significance. The trend in the rate of adoption of the single-transfusion scheme is shown in Fig. 4.

Within the subgroup of patients undergoing liver surgery, a significant reduction in the use of blood transfusion (20.5% vs. 6.7%) and a decrease in the Hb trigger for transfusion (8.5, 8.2–9.5 vs. 8.2, 7.7–8.4 g/dl; *p* = 0.039) was reported after the implementation of the PBM protocol; similarly, a significant decrease in post-operative stay was reported.

## Discussion

Blood transfusion is commonly used as a life-saving treatment in surgical patients, although several negative effects on the early and long-term outcomes have been demonstrated [6, 8, 19].

In the last 10 years, several guidelines and recommendations have been reported to improve the pre-, intra-, and post-operative management of patients with the aim of reducing the use of blood products [12, 14, 20].

In 2015 the Italian Ministry of Health published a recommendation to apply the PBM protocol in all Italian hospitals [21].

In our City Hospital, which recently became an HPB hub [22] for the region, a PBM program was introduced in 2018. We have demonstrated a progressive and significant reduction in blood transfusion since the implementation of this protocol, including a more restrictive use of peri-operative transfusion and the use of single units of packed red cells. In particular, we reported a relevant reduction in the use of packed red cells in gastric, pancreatic, and liver surgery. In contrast, we did not identify any difference in transfused patients treated for colorectal tumors between the two periods. However, the incidence of transfused patients after colorectal surgery was low, even in the first period (only 9%); therefore, demonstration of a possible impact of the new policy was difficult. In colorectal surgery, only 10–15% of patients required blood products [23, 24]; however, in a large series such as in our experience, PBM appeared not to reduce this percentage significantly.

In a large series from the national database, 30% of patients requiring hepatic and pancreatic surgery were transfused [1, 2]. However, in North America, several multicenter studies have demonstrated the possibility of reducing the use of blood products after different types of surgery, even in hepatobiliary and pancreatic surgery [15, 20, 25, 26]. We have demonstrated that in both gastric and liver surgery, only 13.5% and 6.7% of patients, respectively, required intra- or post-operative blood transfusion with the precise application of PBM. Even in pancreatic surgery, after PBM implementation, only 19% of resected patients required transfusion. Similar results have been reported in a recent large study using the US nationwide database; even after liver resection, 30% of patients received blood transfusion in the most recent period [17].

We noted a significant increase in post-operative complications in patients receiving two or more transfusions compared to those requiring only one packed red cell unit [8]. Other authors have reported similar data [19]. Thus, in the last 5 years, a strong recommendation to restrict the use of transfusion has been reported. The strategy of transfusing only one unit of packed red cells increased in all types of surgery without any adverse effects on the post-operative outcomes; this tendency reached a significant difference, particularly in patients requiring gastric and liver surgery.

Recently, a tendency to restrict the use of blood transfusion with a trigger point of hemoglobin of only 7 g/dl has been reported [12, 15]. However, concerns exist that an overly restrictive transfusion strategy may impact post-operative outcomes and increase cardiac complications [27, 28]. Thus, one of the most recent recommendations is to use 8 g/dl as a trigger cut-off for blood transfusion [29]; this has also been included in our protocol in common clinical conditions (e.g., coronary artery disease…) since 2018. In this series, we have reported a median trigger point for blood transfusion of approximately 8.5 g/dl; however, we demonstrated a significant reduction in the trigger point for transfusion after PBM implementation in patients requiring liver surgery (from 8.5 to 8.1 g/dl from the first to the second period).

In the last 20 years, minimally invasive surgery has increasingly been used. Both laparoscopic and robotic surgery have demonstrated several advantages over “open” surgery; among these, the reduction of the intraoperative blood loss appears consistent [30–34]. In the present series, a significant increase in the use of the minimally invasive approach was evident, from 31 to 38% in the first and second periods, respectively. This strategy, which is also recommended in the PBM protocol, may be useful in reducing the use of transfusions.

This study has a few limitations mainly linked to its retrospective design. As such, few variables about specific comorbidities and detailed postoperative complications which could have potentially helped in analyzing the influence of the PBM protocol could not be retrieved. In addition, the number of patients who were transfused per procedure type was relatively low and this might have affected the statistical analysis in the attempt to highlight the impact of the use of the PBM protocol. To overcome these limitations, we believe a multicenter prospective trial may be helpful in confirming the utility of the PBM protocol in limiting the use of red blood cells in patients undergoing HPB and gastrointestinal surgery.

In conclusion, after the implementation of a PBM protocol in a City Hospital, a significant reduction in the number of patients receiving blood transfusion was demonstrated, with a strong tendency to minimize the use of blood products for most types of oncologic surgery. Additionally, a tendency to reduce the trigger cut-off for transfusion and to increase the frequency of use of only one packed red cell unit was reported.
